# Recent developments in the production of prebiotic fructooligosaccharides using fungal fructosyltransferases

**DOI:** 10.1080/21501203.2024.2323713

**Published:** 2024-04-02

**Authors:** Hemant Kumar Rawat, Suresh Nath, Isha Sharma, Naveen Kango

**Affiliations:** Department of Microbiology, Dr. Harisingh Gour Vishwavidyalaya (A Central University), Sagar, MP, India

**Keywords:** Fructooligosaccharides (FOS), fructosyltransferase (FTase), fungi, prebiotic, immobilisation, probiotic

## Abstract

Prebiotic nutritional ingredients have received attention due to their health-promoting potential and related uses in the food and nutraceutical industries. Recent times have witnessed an increasing interest in the use of fructooligosaccharides (FOS) as prebiotics and their generation using microbial enzymes. FOS consumption is known to confer health benefits such as protection against colon cancer, improved mineral absorption, lowering effect on serum lipid and cholesterol concentration, antioxidant properties, favourable dietary modulation of the human colonic microbiota, and immuno-modulatory effects. Comparative analysis of molecular models of various fructosyltransferases (FTases) reveals the mechanism of action and interaction of substrate with the active site. Microbial FTases carry out transfructosylation of sucrose into fructooligosaccharides (kestose, nystose, and fructofuranosylnystose), the most predominantly used prebiotic oligosaccharides. Furthermore, FOS has also been used for other purposes, such as low-calorie sweeteners, dietary fibres, and as the substrates for fermentation. This review highlights the occurrence, characteristics, immobilisation, and potential applications of FOS-generating fungal FTases. Production, heterologous expression, molecular characteristics, and modelling of fungal FTases underpinning their biotechnological prospects are also discussed.

## 1. Introduction

**Figure 1.**
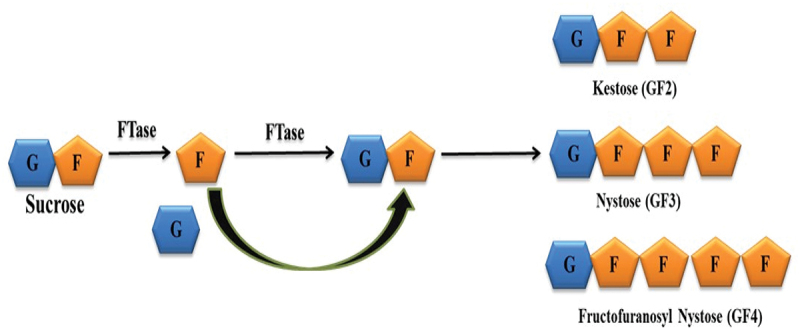
Schematic showing the formation of fructooligosaccharides (FOS) by the action of fructosyltransferase (FTase). FTase cleaves the sucrose molecule and then transfers the fructose residue to other sucrose molecules (G-glucose; F-fructose).

Fructooligosaccharides (FOS) are widely employed prebiotic nutraceuticals. The global prebiotic market has reached USD 6.0 billion in 2022 and is expected to grow to USD 13.8 billion by 2030 (Mano et al. 2018; Rahim et al. 2021). In 2022, the world FOS market was worth USD 2.59 billion. It is further expected to grow at a compound annual growth rate (CAGR) of 8.8% up to 2030 and reach USD 5.09 billion. Owing to its increasing use in beverages, drugs, feed, nutraceuticals, and infant formula foods, the demand for FOS is increasing. The major demand for FOS comes from Europe which shares 29.1% of the market size (https://www.grandviewresearch.com/industry-analysis/fructooligosaccharides-market). Nutraceutical FOS is a mixture of oligosaccharides [1-kestose (GF2), nystose (GF3), and 1-fructofuranosylnystose (GF4)] composed of fructose and glucose. These oligosaccharides have been recognised to selectively stimulate the growth of prebiotic bacteria such as Lactobacilli and Bifidobacteria along with enhanced calcium and magnesium absorption in the large intestine. Short-chain FOS are being used as functional food and feed ingredients in Europe, America, and Asia due to their Generally Regarded As Safe (GRAS) status and have received considerable attention in the nutraceutical sector (Martins et al. 2019; Nath and Kango 2022; Mahalak et al. 2023; de Carvalho Correa et al. 2024). The term FOS is used for fructose oligomers which contain one glucose unit and 2–10 units of 2,4-D-fructose joined together by β-(2, 1) glycosidic linkages (Figure 1). Two classes of fructosylating enzymes are particularly useful for FOS generation at the industrial level; fructosyltransferases (FTases: EC 2.4.1.9) and β-fructofuranosidases (FFases or invertases: EC 3.2.1.26). Fungi (e.g., *Aspergillus* sp. and *Aureobasidium* sp.) are the most prominent producers of FTases employed for the production of FOS at an industrial scale. FTases have higher transfructosylating potential than invertase to convert sucrose into FOS.

Prebiotics are an important intervention from the point of view of their role in ameliorating health conditions by enriching probiotic microbiota, thereby avoiding or minimising the use of antibiotics. Indiscriminate and rampant use of antibiotics has led to the emergence of multi-drug resistance in pathogens. The use of synbiotics (prebiotics and probiotics) can help avoid or limit the use of antibiotics and, therefore, is being looked upon as a promising alternative.

FOS confers beneficial effects on human health by preventing cardiovascular diseases (reduces cholesterol), colon cancer, and osteoporosis *via* increased absorption of Ca^2+^ and Mg^2+^ ions (Sánchez-Martínez et al. 2020; Choukade and Kango 2021; Karkeszova and Polakovic 2023). FOS occurs naturally in various fruits and vegetables such as apples, bananas, leeks, garlic, chicory, artichoke, asparagus, dandelion, onion, agave, tomato, stevia, etc. (Rawat et al. 2017; Rahim et al. 2021). FTases catalyse the transfer of fructosyl unit from sucrose to another acceptor molecule and are produced by a variety of organisms including filamentous fungi (Aspergilli and Penicillia), yeast (*Rhodotorula* and *Candida*), bacteria (Lactobacilli) and plants (*Helianthus* and *Cichorium*) (Sun et al. 2020). Apart from having prebiotic functional properties, FOS is also used as a low-calorie, non-cariogenic sweetener (Liu et al. 2020). A substrate that is selectively utilised by microorganisms and confers health benefits to the host is known as a prebiotic (Gibson et al. 2017). Rosa et al. (2021) have comprehensively reviewed the health-promoting effects of prebiotic substances when consumed with dairy products (processed cheese, yoghurts, ice creams, dessert, and whey beverages) and their anti-diabetic, anti-hypertensive, hypolipidemic, immune-stimulatory, and gut health improving properties.

FOS are predominantly used in infant food formulations as they support the growth of probiotics in an infant’s intestine and help in the development of its immune system and overall health (Miqdady et al. 2020). Furthermore, FOS are being incorporated into food, beverages, and ice creams as natural low-calorie sweetener and dietary fibre (Ahmadi et al. 2014). FOS is also being used as a healthy and low-calorie sweetener (Bali et al. 2015). Prebiotic oligosaccharides alter the composition and activity of gastrointestinal tract microflora which, in turn, imparts health benefits to the host. Intake of FOS results in increased numbers of health-promoting Lactobacilli, Bifidobacteria, and *Bacteroides* in the intestine (Ni et al. 2020; Fang et al. 2021). Although FOS has gained centre stage as a functional oligosaccharide and its consumption is predicted to grow, however, the production strategies have not been effective in bringing down the cost. Accordingly, bioprocesses based on novel FTases sourced from robust and hyperproducing fungal strains are to be devised. Also, FOS production using cheaper alternative substrates can help make the process economic. Given the above, it is important to develop bioprocesses with a cost-effective approach for the economical and efficient production of FOS. Microbial biotransformation of cane sugar to FOS using FTase is being explored as a suitable alternative. In this regard, microorganisms are being evaluated for FTase production. In the present review, we discuss the production and properties of FTase-producing microorganisms. Furthermore, purification, immobilisation, molecular characterisation and molecular docking studies of FOS-generating fungal FTases have been discussed. The functional and health-promoting properties of FOS have also been described.

## 2. Properties of FOS-producing microorganisms

FOS can be produced enzymatically by transfructosylation of sucrose by FTase which specifically cleaves a sucrose molecule and then transfers the liberated fructose molecule to an acceptor moiety, i.e. sucrose or another oligosaccharide, for elongation of the short-chain FOS (GF2, GF3, and GF4) (Figure 1). Food-grade sucrose can be converted into FOS by employing transfructosylating enzymes originating from fungi *Aspergillus oryzae*, *Aspergillus flavus*, *Aspergillus japonicus*, *Aspergillus foetidus*, *Aspergillus niger*, *Aureobasidium pullulans*, *Penicillium citrinum*; bacteria *Lactobacillus reuteri*, *Zymomonas mobilis*, and *Bacillus macerans*; and yeasts *Rhodotorula*, *Candida*, and *Cryptococcus*. However, *Aspergillus* remains one of the most explored genera for the production of FTase for efficient FOS generation (Table 1). All the eight *Aspergillus* strains screened by Barros et al. (2020) produced GF2, GF3, and GF4 with sucrose as substrate. A mutant *A. niger* ATCC 20,611 fungal strain was used for the production of FOS (GF2–24.5%, GF3–20.3%, GF4–6.5%) using 50% (w/v) sucrose as the substrate for transfructosylation (Zhang et al. 2019). Optimal FOS production with a yield of 20.30 g/L sucrose was achieved with *A. oryzae* FTase using 42.64 g/L initial sucrose in 24 h of biotransformation. The productivity of sucrose biotransformation was 0.84 g/L (Muñiz-Márquez et al. 2019). Nobre et al. (2019) have reported a maximum FOS yield of 0.55 ± 0.02 g FOS/g initial sucrose (100 g/L) in 7 days of fermentation with a net yield of 3.9 g/L using *Penicillium citreonigrum*. A variety of production methods including submerged and solid-state fermentation (SSF) have been used for FTase-producing fungi (Table 1). The scenario of FTase-producing fungi is dominated by Aspergilli and the major products of transfructosylation are observed to be GF2, GF3, and GF4.

**Table 1.** Microbial production of fructooligosaccharides (FOS).

SN	Source of enzyme	Type	FTase (U/mL)	pH	Temp (°C)	Incubation time (h)	RPM	Substrate	FOS produced (% w/w)	References
1	*Aspergillus niger*	SSF	-	6.5–7.0	40–45	24	150	Sucrose (400 g/L)	32.4 mg/mL	Guerra et al. (2023)
2	*Aspergillus* sp.	SmF	5.0	5.5	55	-	-	Sucrose (2,000 mmol/L)	GF2–300 g/L; GF3–200 g/L; GF4–120 g/L; GF5–42 g/L	Karkeszova and Polakovic (2023)
3	*Aspergillus welwitschiae* FAW1	SmF	-	6.0	60	24	-	Sucrose (60%)	Kestose, nystose	Stojanovic et al. (2022)
4	*Aspergillus oryzae* S719, *Wickerhamomyces anomalus*	SmF	Mycelial FFase	-	50	24	120	Sucrose (300 g/L)	87% (0.63 g FOS/g GF) Purity (FOS 95.6%)	Zeng et al. (2022)
5	*Neocosmospora vasinfecta,* *Fusarium solani*	Smf SmF	--		28 28	14 14	140 140	Sucrose (10 g/L) Inulin (10 g/L)	50 mg/L (1-Kestose) 4 mg/L (6-Kestose) 92 mg/L (1-Kestose)	Galvao et al. (2021)
6	*A. oryzae* DIA-MF	SmF	-	5.5	30	36	200	Sucrose (165 g/L)	119 ± 12 g/L FOS	De La Rosa et al. (2020, 2022)
7	*Aspergillus tritici* BGPUP6	SmF	25	5.5	55	12	150	Inulin (2%)	14.0	Singh et al. (2021)
	*A. tritici* BGPUP6	SmF	50	5.5	55	10	125	Inulin (6%)	97.27	
8	*Zalaria* sp. Him3	SmF	10	5.0	-	72	-	Sucrose (500 g/L)	63.7 (CS-FFase) 64.6 (EC-FFase)	Yoshikawa et al. (2021)
9	*A. niger* ATCC 20611	SmF	50	5.5	50	-	200	Sucrose (50%)	51.0	Wang et al. (2021)
10	*Aureobasidium pullulans* FRR 5284	SmF	20	5.5	55	12	100	Sucrose (500 g/L)	62.7 ± 0.2	Khatun et al. (2020)
11	*A. pullulans* FRR 5284	SmF	-	5.5	55	3	-	Sucrose (50%)	61.0	
12	*A. oryzae* S719	SmF	Mycelium	6	50	20	150	Sucrose (900 g/L)	586.0 ± 4.7 g/L	Han et al. (2020)
13	*A. oryzae* DIA-MF	SSF	-	4.5	30	12	-	Sugarcane bagasse (5 g)	7.64	De La Rosa et al. (2020)
14	*Aspergillus carbonarius,* *Aspergillus japonicus*	SmF	1 mL	5.0	55	24	-	Sucrose (50%)	26.77^I^ and 24.57^E^ 19.18^I^ and 21.34^E^ 21.00^I^ and 20.75^E^	Barros et al. (2020)
15	*Aspergillus fumigatus*	SmF	1–2	5.5	50–60	4	-	Inulin (2%)	4.076 Nystose 5.72 Kestose (mg/mL)	Chikkerur et al. (2020)
16	*A. tritici* BGPUP6	SmF	25	5.5	55	6–24	150	Inulin (4%)	39.98	Singh et al. (2020)
17	*A. niger* ATCC 20611	SmF	6 U/g sucrose	-	50	5	-	Sucrose (50%, w/w)	GF2–24.5%; GF3–20.3%; 6.5% GFn-51.3%	Zhang et al. (2019)
18	*Zymomonas mobilis*	SmF	2.1	5.2	35	6	-	170 g/L	65 g/L	Taştan et al. (2019)
19	*Aspergillus thermomutatus*	SmF	0.015–	5.0	60	72	-	500 g/L	86.7 g/L	Tódero et al. (2019)
20	*A. melanogenum*	SmF	117 U/g	4.5	50	1–10	-	300 g/L	660 g/kg sucrose	Aung et al. (2019)
21	*Komagataella pastoris*	SmF	6	5	60	3–12	-	60%	61.4	Bedzo et al. (2019)
22	*A. oryzae* DIA-MF	SmF	10	4.5	30	10	-	Aguamiel	31.01 g/L FOS	Picazo et al. (2019)
23	*A. oryzae* DIA-MF	SmF	-	4.5	30	24	180	42.64 g/L	20.30 g/L	Muñiz-Márquez et al. (2019)
24	*Aspergillus ibericus*	SmF		6.2	37	38	150	100 g/L	64%	Nobre et al. (2019)
25	*Saccharomyces cerevisiae,* *Rhodotorula mucilaginosa*	SmF	1	4.05	50	2	Static	Sucrose (20%)	13.3 g/L 12.60 g/L	Barbosa et al. (2018)
26	*A. niger* ATCC20611	SmF	6 U/g	5.5	50	3	120	Sucrose	59%	Zhang et al. (2017)
27	*A. oryzae*	SmF	0.85^#^	6.0	60	3	200	800 g/L	480 g/L	Zhang et al. (2016)
28	*A. oryzae*	SSF	-	5.5	32	24	-	37 g/L	22	Muniz-Marquez et al. (2016)
29	*A. niger* ATCC 26011, *Penicillium citrinum* MTCC 1265, *Penicillium rugulosum* MTCC 3487	SmF	1.98 4.64 3.58	5.0 5.0 5.0	30 30 30	72 72 72	150 150 150	20 20 20	12.76 21.20 7.590	Rawat et al. (2015)

SmF, submerged fermentation; SSF, solid state fermentation; FOS, fructooligosaccharides; ^#^, U/mg dry cell weight; EC, culture broth; CS, cell surface; I, intracellular; E, extracellular.

## 3. Purification of FOS-generating microbial enzymes

Purification of an enzyme is important to decipher its prominent and distinguishing characteristics for its successful application. An elaborate account of the purification strategies for fungal FTases and FFases including precipitation (using ammonium sulphate, ethanol, and acetone, etc.), dialysis, ultra-filtration, and a combination of chromatographic techniques is provided in Table 2. FTase from *A. terreus* was purified using ion exchange matrices Q-Sepharose and Phenyl-Sepharose column followed by gel filtration matrix, Sephacryl S-300. The purified FTase was a homodimeric protein composed of two 32 kDa units and displayed maximum transfructosylation at 4.5 pH and 55 °C. The enzyme had a *K*_*m*_ of 6.2 mmol/L for sucrose and a half-life of 2 h at 60 °C (de Almeida et al. 2018). FTase from *Aspergillus tamarii* was purified by a two-phase aqueous polyethylene glycol/citrate system. The FTase had a molecular weight of 66 kDa and was optimally active at 55 °C (Batista et al. 2021). Han et al. (2021) have purified *Aspergillus oryzae* extracellular FTase using a combination of precipitation and chromatographic techniques, *viz*. ammonium sulphate precipitation, membrane filtration, DEAE-Sepharose, and Sephacryl S-200 HR. The optimum pH and temperature were 55 °C and 6.0, respectively. Choukade and Kango (2022) have purified *A. tamarii* mycelial fructosyltransferase (*m*-FTase) using ultrafiltration followed by HiTrap Q HP anion exchange chromatography. The 2.15-fold purification was achieved with the 75 kDa FTase showing 12.76 U/mg specific activity. The extracellular FTase of *A. oryzae* S719 was purified using a combination of Sephacryl S-200 and DEAE-Sepharose chromatography. FTase (95 kDa) was optimally active at 55 °C and pH 6.0 (Han et al. 2020). Batista et al. (2020) have purified FTase from an *A. tamarii* Kita UCP1279 by ethanol precipitation (70%), SOURCE 15Q (anionic exchanger) and Superdex-G75 (size exclusion chromatography). After purification, FTase (89.7 kDa) was used for FOS production.   Ojwach et al. (2020) have reported Mg^2+^, K^+^, Ca^2+^ to enhance the *A. niger* XOBP48 FTase activity, while Hg^2+^ and Ag^+^ at 1 mmol concentration inhibited the enzyme.

**Table 2.** Properties and purification strategies of FOS generating microbial enzymes (FTase/FFase).

SN	Organism	Type	MW (kDa)	pH	Temp (°C)	*K*_*m*_ (mmol/L)	*V*_*max*_ (µmol/min)	*K*_*cat*_ (min^−1^)	*K*_*cat*_*/K*_*m*_ (µmol^−1^ min^−1^)	Activators	Inhibitors	Purification strategy	Reference
1	*Aspergillus tamarii* NKRC 1229	FTase	75	7.0	20	1,049.7	2.094	-	-	Cu^2+^	Hg^2+^, Mn^2+^	Ultrafiltration, HiTrap Q HP Anion Exchange Chromatography	Choukade and Kango (2022)
2	*A. tamarii* Kita	FFase	66	5.15	55	42.9	180.2	-	-	CaCl_2_, ZnCl_2_, ZnSO_4_	FeCl_3_, FeSO_4_	Two-phase aqueous PEG/citrate system, polyethylene glycol (50% w/w), sodium citrate (30% w/w)	Batista et al. (2021)
3	*Aspergillus oryzae* S719	FTase	95	6.0	55	310	1.4	2.0 × 103	6.4	Mg^2+^, Na^+^, Ca^2+^	Fe^2+^, Pb^2+^	Ammonium sulphate, Membrane filtration, DEAE-Sepharose, Sephacryl S-200 HR	Han et al. (2021)
4	*A. tamarii* Kita UCP1279	FTase	89.7	5.0	60	-	-	-	-	No activator	CuSO_4_, FeSO_2_, FeCl_3_	Ethanol precipitation (70%), SOURCE 15Q (anionic exchanger), Superdex-G75	Batista et al. (2020)
5	*Aspergillus niger* XOBP48	FTase	70	6.0	50	79.51	45.04	31.5	396	Mg^2+^, K^+^, Ca^2+^	Hg^2+^, Ag^+^	Ammonium sulphate, Dialysis, anion exchange chromatography	Ojwach et al. (2020)
6	*Aureobasidium melanogenum*	FFase	82.4	4.5	50	142	1.214 mol/min	-	-	Ca^2+^, Mn^2+^, Fe^2+^, K^+^, Cu^2+^, Co^2+^, Ba^2+^	Mg^2+^, Zn^2+^, PFMS, SDS, DTT	Dialysis, DEAE sepharose, Ultrafiltration (10 kDa), Sephadex S-100	Aung et al. (2019)
7	*Aspergillus terreus*	FTase	32 34	4.6 3.0	55 60	9.8 6.2	-	-	-	No activator	AgNO_3_, Mg^2+^, Ca^2+^, CuSO_4_, Cu^2+^, SDS	Ultrafiltration (30 kDa), Ion exchange (Q-Sepharose), Hydrophobic interaction chromatography (Phenyl-Sepharose column), Sephacryl S-300	de Almeida et al. (2018)
8	*A. niger* SG610	FTase	120	5.5	50	424.5	5.9	3.8 × 10^3^	9.0	Mg^2+^, Fe^2+^	Li^+^, Al^3+^, Zn^2+^, Ag^+^, Cu^2+^	6 His-tagged recombinant FruSG, Ni^2+^ column	Guo et al. (2016)
9	*A. oryzae* ZZ-01	FTase	50	6.0	45	-	-	-	-	Mg^2+^, K^+^, Propanol, Toluene, Tween20, Triton, X-100		Ammonium sulphate, Q- Sepharose FF, Phenyl sepharose, Sephacryl S-200 HR	Wei et al. (2014)
10	*Xanthophyllomyces dendrorhous* Xd-INV (a and b)	FFase a b	200 160	4.56 4.56	65–80 65–75	4.1 3.7	0.065 0.051	422,313	100 84	-	-	MWC PEC membrane, Dialysis, DEAE-Sephacel	Linde et al. (2012)
11	*Rhodotorula* sp.	FTase		4.5	50	428.9	5.8	-	-	-	-	Ethanol precipitation, Q- Sepharose, Ultrafiltration	Alvarado-Huallanco and Maugeri-Filho (2011)
12	*X. dendrorhous* 269	FFase		6.4	45	511 mmol/L	233 µmol/min mg)			Ca^2+^, Li^+^, Mn^2+^, Al^3+^, Ba^2+^, EDTA	Mg^2+^, K^+^, Zn^2+^	DEAE-52 Cellulose	Chen et al. (2011)
13	*Rhodotorula dairenensis*	FFase	172	5.0	55–60	1.2	-	6.5	5.4	-	-	MWCO PES (30,000), DEAE-Sephacel	Gutierrez-Alonso et al. (2009)
14	*Aspergillus aculeatus*	FTase	85	6.0	60	272.3	166.7	-	-	-	-	Dialysis, Polyethyleneglycol (30%), DEAE-Sephacel	Nemukula et al. (2009)

FTase, fructosyltransferase; FFase, fructofuranosidase.

## 4. FOS generation using immobilised biocatalyst

In recent years, immobilisation has received worldwide attention as it renders the enzyme reusable, provides greater stability and catalytic control, prevents product contamination, allows continuous product formation, and thus, has great potential for industrial applications. Reusability of the immobilised enzymes also increases cost-effectiveness and helps in the actual realisation of FTase potential in prebiotic FOS synthesis. Immobilisation of whole cells and FTase/FFase has been extensively used to catalyse continuous FOS generation using high concentrations of sucrose or inulin as substrate (Table 3). Chitosan and alginate served as a good matrix to entrap mycelial FTase for the successful production of FOS. Mycelial FTase of *A. flavus* NFCCI 2364 was immobilised in calcium alginate and chitosan support by gel entrapment with a maximum yield of FOS 62.96% (w/w) was reported using FTase entrapped alginate beads. End products formed effectively during recycling (15 cycles) of immobilised enzymes were GF4, GF3, GF2, G, and F (Ganaie et al. 2014). *A. oryzae* IPT-301 FTase was immobilised using alginate beads with glutaraldehyde as a cross-linking agent. The immobilised FTase was reusable up to 3 cycles of 4 h each with residual activity of 92% (Gonçalves et al. 2020). *A. pullulans* NAC8 FTase was used to produce cross-linked enzyme aggregates (CLEAs) using 5% (v/v) glutaraldehyde. CLEAs were active up to 6 reaction cycles with 70% of the residual activity (Ademakinwa et al. 2018). *A. aculeatus* FTase was immobilised in gelatin gel and then cross-linking by treatment with glutaraldehyde with an immobilisation yield of 55%. The optimum pH and temperature were 5.5 and 55 °C for the immobilised enzyme, respectively (Lorenzoni et al. 2014). Choukade and Kango (2019) have used the mycelial FTase of *A. tamarii* NKRC 1229 for continuous generation of FOS in a Poly-Prep chromatography column to obtain GF3 and GF2. Continuous production of FOS (GF2 and GF3) from molasses as substrate using *Sclerotinia sclerotiorum* FFase immobilised covalently on alginate and chitosan resulted in a yield of 72.2% FOS/g sucrose in 12 h. The enzyme was stable over a wide pH (4.0–7.0) and temperature (4–70 °C) range (Mouelhi et al. 2016).

**Table 3.** Enzymatic and whole-cell immobilisation for continuous FOS generation.

SN	Microorganism	Sucrose (w/v)	Optimum		Carrier	Yield (% w/w sucrose)	FOS products	Recycling	Reference
			Temp (°C)	pH					
1	*Aspergillus tritici* BGPUP6	Inulin (7.0%)	-	-	Halloysite nanoclay	99.56% FOS	GF4, GF3, GF2, and FOSs DP5–9	3 cycles	Singh and Singh 2022a, 2022b)
2	*A. tritici*	Inulin (8.0%)	60	5.0	Halloysite nanoclay	95.44% FOS	GF4, GF3, and GF2	8 cycles	Singh and Singh (2022b)
3	*A. tritici*	Inulin (10%)	65	5.0	Halloysite nanoclay using 3-amino-propyltriethoxysilane	98.42% FOS	GF4, GF3, GF2 and FOSs DP 5–9	18 cycles	Singh and Singh (2022c)
4	*Aspergillus oryzae* IPT-301		30		Polyhydroxybutyrate	55% FOS		6 cycles	Araújo et al. (2022)
5	*Penicillium brevicompactum* FTase in *Pichia pastoris* GS115 (Muts)	Sucrose (600 g/L)	45	6.0	Lifetech ECR8285 (methacrylate polymer)	130–170 g/L	GF4, GF3, and GF2	-	Fang et al. (2021)
6	*Aspergillus niger* ATCC 20611	Sucrose (50%)	50	5.5	Mycelia of *A. niger*	51.05% FOS	-	6 cycles	Wang et al. (2021)
7	*Schedonorus arundinaceus* (*P. pastoris*)	600 g/L	30	5.5	Calcium-alginate	55% FOS	1-Kestotriose and 1,1-Kestotetraose	15 cycles	Pérez et al. (2021)
8	*Rhodotorula mucilaginosa*	Inulin 250 g/L (25%)	75	8.0	Celite and chicken eggshell	9.49 g/L	GF3 GF2	16 h	de Araujo Ribeiro et al. (2022)
9	*A. oryzae* IPT-301	47	60	5.5	Silica gel	-	GF2	6 cycles	Faria et al. (2021)
10	*Aspergillus aculeatus*	700 g/L	60	7.0	Fe_3_O_4_-Chitosan- magnetic nanoparticles	101.56 g/L	GF3, GF2	6 cycles	de Oliveira et al. (2020)
11	*Aspergillus terreus*	1.4–1.9 Molar	70	5.8	Sepabeads SP70, Amberlite XAD16N, and immobead 150P carriers	60% FOS yields	GF5, GF4, GF3, and GF2	-	Burghardt et al. (2020)
12	*Aspergillus tamarii* NKRC 1229	50	20	7.0	Poly-Prep chromatography column	50%	GF3, GF2	10 cycles	Choukade and Kango (2019)
13	*Aureobasidium* sp. ATCC 20524	100, 300, 600 g/L	50	5.0	Titanium oxide	60, 59, 62	GF4, GF3, and GF2	7 cycles	Valdeon et al. (2019)
14	*Aspergillus japonicus* (*fopA*)	60%	62	5.0	Amberlite IRA 900 and calcium alginate	53% and 59% FOS	GF4, GF3, and GF2	15 cycles	Bedzo et al. (2019)
15	*Xanthophyllomyces dendrorhous*	600 g/L	30	5.0	Polyvinyl alcohol hydrogel	18.9% FOS yields	Neokestose, 1-Kestose, neonystose, blastose	7 cycles	Miguez et al. (2018)
16	*Aspergillus aculeatus* M105	600	65	5.0–6.0	Sodium alginate	65.47	GF4, GF3, and GF2		Huang et al. (2016)
17	*A. aculeatus*	600	50	5.5	Chitosan	55	GF3, GF2	50 cycles	Lorenzoni et al. (2014)
18	*Aspergillus flavus* NFCCI 2364	60	55	5.5–6.0	Calcium alginate, chitosan	68 43	GF4, GF3, and GF2	15 cycles	Ganaie et al. (2014)
19	*Aureobasidium pullulans*	770	50	-	Calcium alginate	57	GF4, GF3, and GF2	100 days	Jung et al. (2011)
20	*A. japonicus*	165 g/L	-	-	Brewers spent grain, wheat straw, corn cobs, coffee husks, cork oak, loofa sponge	128.3–138.7 g/L	GF4, GF3, and GF2	36 h	Mussatto et al. (2009)

## 5. Cloning and heterologous expression of FOS generating organisms

Recently, there has been a spurt of interest in finding novel FTase and FFase producers and cloning and expression of these genes in heterologous hosts (Chu et al. 2022). Over the last decade, several successful attempts have been made to clone and express the FTase/FFase genes from distinct hosts including moulds, yeasts, and bacteria (Table 4). The *ftase* gene of *A. oryzae* N74 had an open reading frame of 1,630 bp with 99% similarity with other *A. oryzae* G×0015 and RIB40, encoding 525 amino acids (99% similarity with *A. oryzae*) and a conserved DNA (>18 bp) and protein (>5 amino acids). The recombinant purified FTase had a molecular weight of 57 kDa, with a signal peptide sequence of 17 amino acids. Structure elucidation for understanding enzyme action and protein engineering is a recent approach in biocatalysis. Structural and computational studies showed that FTase has 4.83 isoelectric point (pI) and 6 glycosylation sites. *A. oryzae* FTase tertiary structure was modelled by two different modelling tools. In the GENO3D (homology-based modelling tool) modelling FTase crystal structure was very similar to *Thermotoga maritima* β-fructosidase (PDB No. 1 UYP) which showed 29.2 identical similarities and in the case of the PHYRE tool (threading-based tool for tertiary structure prediction) showed the lower E-value zero and 31% identity with the exoinulinase of *A. awamori* (PDB No. 1 Y9G) (Rodriguez et al. 2011). Expression of the *A. oryzae* FS4 β-fructosidase gene was performed in *E. coli* and *Pichia pastoris*. The protein deduced from the cloned BfrA contained signal peptide which had an N-terminal sequence (88% similarity) and amino sequences (90% identity) with *A. oryzae* RIB40 and *A. flavus* NRRL3357 extracellular invertases. Purified BfrA (70 kDa deglycosylated) had 13 N-glycosylation sites (Asn-X-Ser/Thr) and produced levan and neolevan type FOS. The composition of FOS was analyzed by Bio-Gel P2, TLC, and HPLC and also characterised by mass and NMR analysis (Li et al. 2014). Wei et al. (2016) developed recombinant BL 21-Codon Plus (DE3)-RIL expressing FTase from *A. oryzae* ZZ-1 resulting in 38 times higher enzyme yield as compared to the native strain. β-FFase from *Aureobasidium melanogenum* 11-1 was used for cloning and overexpression for high FOS generation. The 2.3 kb FFase gene had the conserved domain A (IGDP), domain (RDP), and domain E (ET) and 11 N-glycosylation sites. The recombinant FFase from *A. melanogenum* transformant 33 has resulted in 0.63 g of FOS yield per gram of sucrose in 5 h (Aung et al. 2019). Burghardt et al. (2020) have edited the native *Aspergillus terreus* invertase (fructofuranosidase) gene using CRISPR/Cas9 genome editing tool and found higher fructosyltransferase activity of mutant (66.9%) as compared to native strain (49.7%) leading to increased FOS production.

**Table 4.** Heterologous expression and characteristics of cloned fructosyltransferases (FTases).

SN	Organism	Gene (ORF)	Expression Host	MW (kDa)	pH	Temp (°C)	Properties of cloned enzyme	FOS	Reference
1	*Aspergillus oryzae* N74	*ftase*	*Komagataella phaffii* (*Pichia pastoris*)	85	5.5	60	508 aa, site-directed mutagenesis	GF4, GF3, and GF2	Alvarado-Obando et al. (2022)
2	*Schwanniomyces occidentalis*	sacB	*Zymomonas mobilis* ZM4	47	-	-	Levan sucrase in *Z. mobilis* ZM4	1-GF2, 6-GF2, and GF3	Braga et al. (2022)
3	*Aspergillus japonicus*	Aj-FTase	*Yarrowia lipolytica*	-	-	-	Heat resistance	GF4, GF3, and GF2	Zeng et al. (2022)
4	*S. occidentalis* ATCC 26077	FTase*inv*	*S. cerevisiae* BY4741 YIL162W SUC2	95	4.5	-	6-kestose producing enzyme	6-GF2 (76 ± 3 g/L), 1-GF2 (1.6 ± 0.6 g/L)	Amorim et al. (2022)
5	*Aspergillus niger* SG610	FruSG	*K. phaffii* GS115	-	5.5	50	Good kinetic parameters of the mutants	65% FOS	Xia et al. (2022)
6	*Aureobasidium melanogenum*	β- FFase 1	*A. melanogenum*	82.4	4.5	50	Over expression of β-FFase 1 with 557.7 U/mL activity	GF2, GF3, and GF4	Amorim et al. (2022)
7	*A. niger* ATCC 20611	FFase fopA-V1	*Escherichia coli*, DH5α	-	5.0	50	91.2% activity after incubation at 50 °C for 30 h	GF4, GF3, and GF2	Wang et al. (2021)
8	*A. niger* FV1-11	*fopA*-V1	*A. niger* ATCC 20611	-	5.5	50	Recycling of mycelia upto 6 cycles with 55% FOS production in each cycle	FOS	Han et al. (2020)
9	Asparagus racemosus*	*aoft3*	*P. pastoris*	70.07	-	-	Nystose producing from 1-kestose	DP ≥ 6	Ueno et al. (2020)
10	*Aspergillus fijiensis*	GAP fopA_V1	*P. pastoris*	-	5.0	-	54.94% FOS in 20 L fermenter using 60% sucrose	GF2, GF3, and GF4	Coetzee et al. (2020)
11	*A. niger* ATCC 41686	(FT-A)	*P. pastoris*	116	6.0	50	Novel fructosyltransferase (FT-A)	GF2, GF3	Mao et al. (2019)
12	Schedonorus arundinaceus*	*Sa1*-SST	*P. pastoris*	-	5.0–6.0	45–50	It produced 55%–60% (w/w) sc-FOS	GF2, GF3	Hernández et al. (2018)
13	*A. niger* YZ59	FTase	*P. pastoris*	-	5.5	55	*K*_*m*_ 159.8 g/L, *V*_*max*_ (µmol/min/mg) 0.66 (g/L min)	GF4, GF3, and GF2	Yang et al. (2016)
14	*A*. *oryzae* ZZ-01	FTase	*E. coli*	60	5.5	55	*K*_*m*_ −21, *V*_*max*_-75 (r-AoFT)	Short-chain sucrose (sucrose 6-acetate, glucose 6-acetate)	Wei et al. (2016)
15	*A. oryzae* FS4	FFase *bfrAFS4* (1,860 bp)	*E. coli* *P. pastoris*	95	4–11	55	*K*_*cat*_-1.1 × 10^4^ min^−1^, *K*_*cat*_*/K*_*m*_ 269.34 mmol^−1^s^−1^ 21	GF4, GF3, and GF2	Li et al. (2014)
16	*Ceratocystis moniliformis*	FFase (*cmINY*)	*S. cerevisiae* BY4742 Suc 2	66 (615 aa)	6	62.5	*K*_*m*_- 7.50, *V*_*max*_-986 (µmol/min/mg)	GF3, GF2	Wyk et al. (2013)
17	*A. oryzae* N74	FTase (*ftase*gene) 1,630 bp	-	57 (525 aa)			PI-4.83	-	Rodriguez et al. (2011)

*FTase from plant source was cloned in yeast.

## 6. Molecular characterisation and modelling of some prominent FTases

**Figure 2.**
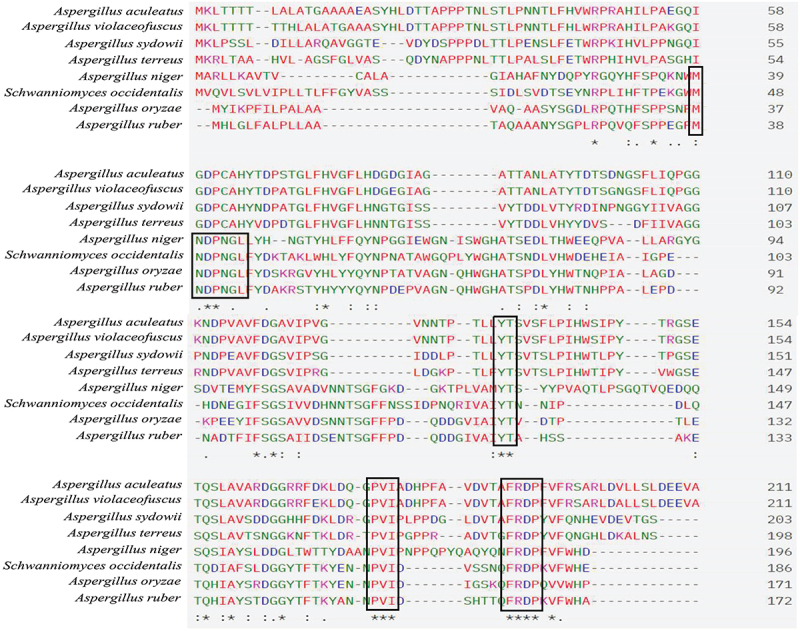
Multiple sequence alignment of fructosyltransferases (FTases) of some fungi. The highlighted section represents conserved sequences MNDPNGL, FRDP, PVI, and YTS among others.

**Figure 3.**
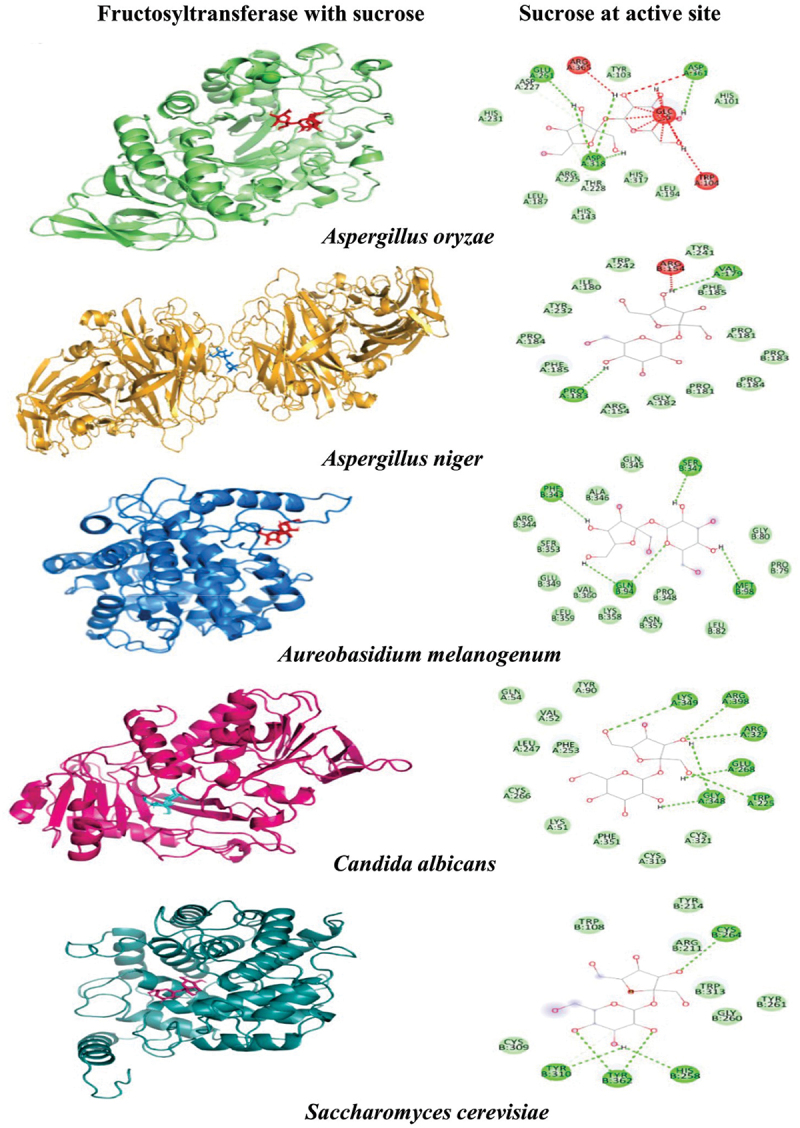
Conserved motifs and structural details of different fungal fructosyltransferases (FTases).

Alignment of some prominent Aspergilli FTase amino acid sequences revealed some of the conserved sequences in their structure (Figure 2). Earlier also, Choukade and Kango (2021) observed that WMNDPNG, FRDP, and ECP are the conserved sequences among FTases. Glycoside hydrolase (GH) 32 family enzymes (FTases) are collectively placed in the clan-J group due to similarities in their tertiary structure. Alignment studies confirmed the related conserved sequences present in related genera. Most of the structural studies suggested the occurrence of different conserved domains in related members. The active site of *A*. *niger* FTase contained specific amino acids (glutamic acid, isoleucine, and cysteine) which are present in the active centre for the fructosylation catalytic mechanism (Xia et al. 2022). A homology-based model of FTases from different fungal strains was used to create FTase structures interacting with sucrose molecules. Different homology models were created for *A. oryzae*, *A. niger*, *A. melanogenum*, *Candida albicans*, and *Saccharomyces cerevisiae* and *in silico* docking with sucrose was used to delineate the catalytic interaction. The electrostatic surface potential of the models was used to observe potential FTase: Sucrose interactions. Further, molecular dynamic analysis was done for FTase docked with substrates and Autodock-4.2 was used to perform rigid docking with fixed ligand size models and flexible ligands sucrose. The representation of the molecule within the active site represents the orientation with the least binding energy conformation. The details of the FTase models after docking and molecular dynamics are presented in Figure 3. Recently, Alvarado-Obando et al. (2022) performed *in silico* study of *A. oryzae* N74 FTase, and the study revealed that positively charged arginine amino acid plays a key role in the catalytic domain. Furthermore, this study predicted that amino acids (Q57, G66, Q68, Y96, F98, D127, K160, and W305 or conserved amino acids) interact specifically with sucrose and GF2 in most of the docking interactions. Docking studies provided some of the catalytic differences between fungal FTases. The electrostatic surface potential of the models was used to observe potential interactions between the enzyme and the substrates.

## 7. Applications

**Figure 4.**
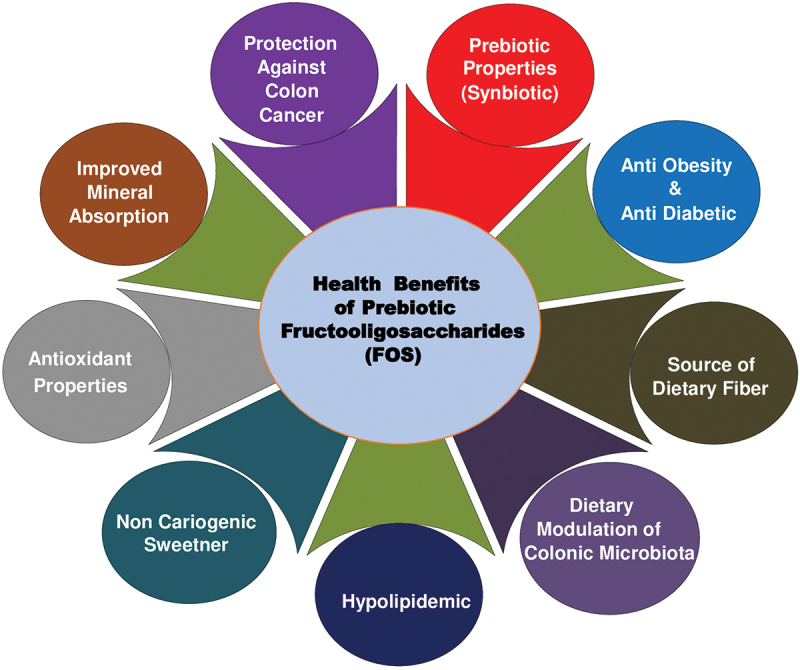
Health benefits of prebiotic fructooligosaccharides (FOS).

Fructo-oligosaccharides (FOS) generated from sucrose using fungal FTases have several functional properties such as antineoplastic, antidiabetic, antioxidant, mineral absorption, and Bifidus-stimulating activities (Figure 4) (Choukade and Kango 2021).

### 7.1. Prebiotic properties of FOS

**Figure 5.**
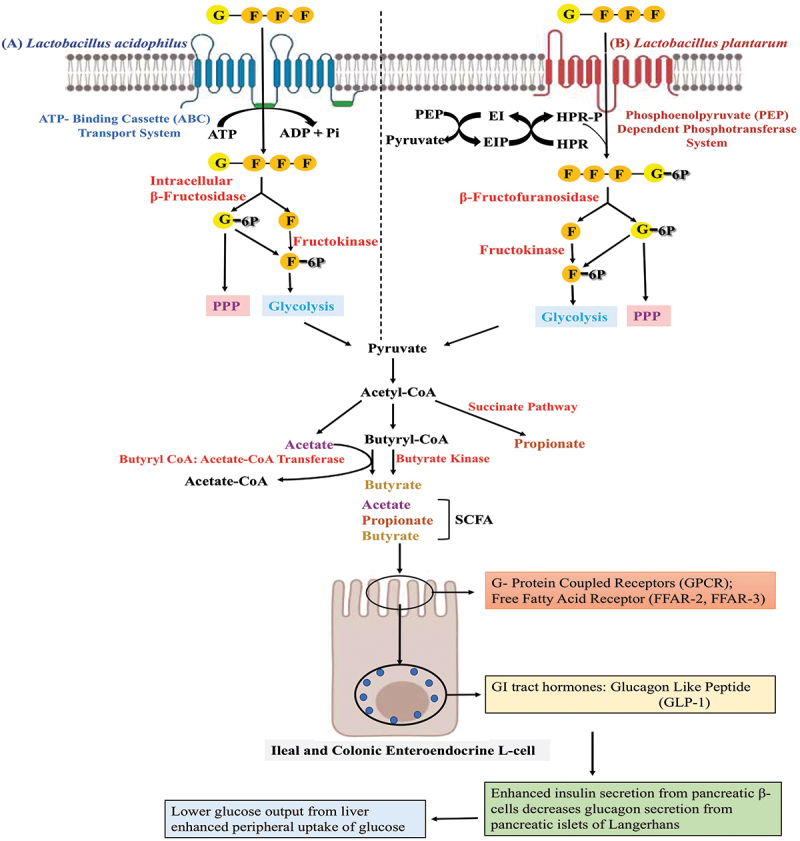
Fructooligosaccharide (FOS) uptake and metabolism by (A) *Lactobacillus acidophilus* and (B) *Lactobacillus plantarum* showing FOS fermentation leading to the production of short-chain fatty acids (SCFA). Absorption of SCFA by ileal and colonic enteroendocrine L-cell and further effects in metabolic regulation. ATP, adenosine triphosphate; ADP, adenosine diphosphate; PPP, pentose phosphate pathway; E1, enzyme 1; HPR, histidine protein; G, glucose; F, fructose; PPP, pentose phosphate pathway; P, phosphate; Pi, inorganic phosphate.

Prebiotic oligosaccharides such as FOS serve to enhance the production of beneficial metabolites, especially short-chain fatty acids (SCFA), in the colon by intestinal microflora. These feed supplements are utilised by beneficial microorganisms and potentially reduce colonisation of food-borne pathogens (Ashaolu 2020; Rahim et al. 2021). *Lactobacillus acidophilus* and *Lactobacillus plantarum* (Chen et al. 2015) cell membranes have ABC transporter (Tsujikawa et al. 2021) and phosphoenol pyruvate-dependent phosphotransferase system, respectively, for intake of FOS inside the cytoplasm. Following intake, the intracellular β-fructosidase hydrolyzes FOS into simpler forms and it is utilised in glycolysis for the generation of pyruvate. Pyruvate is further used in the formation of short-chain fatty acids (SCFA) using different pathways and these SCFA are released into the intestinal lumen where they lower the pH and modulate gut microflora. Also, the SCFA absorbed by intestinal villi of ileal and colonic enteroendocrine L-cells causes secretion of the anorexigenic hormones peptide YY (PYY) and glucagon-like peptide 1 (GLP-1) for metabolic regulation (O’Riordan et al. 2022). The role of probiotic Lactobacilli in the formation of SCFA and the action of SCFA are explained in Figure 5.

Administration of *Lactobacillus salivarius* UBL S22 and prebiotic FOS for six weeks led to a significant reduction in low-density lipoprotein (LDL) cholesterol, triglycerides and it helped increase the high-density lipoprotein (HDL) level due to the combined action of synbiotic (Rajkumar et al. 2015). Prebiotics effectively increased cytokine secretion [tumour necrosis factor alpha (TNF-α), interleukin (IL)-6, and IL-10] in mouse splenocytes but inhibited LPS-induced interferon-gamma (IFN-γ) and IL-17 release. It is shown to induce the production of a growth-related oncogene (GROα), monocyte chemoattractant protein (MCP), and macrophage inflammatory protein (MIP) in IEC18 cells (Capitán-Cañadas et al. 2014). FOS supplementation in the male rats significantly increased the SCFA levels (1,151 ± 108 μg/g) including acetic acid (591 ± 140 μg/g), propionic acid (335 ± 189 μg/g), and butyric acid (225 ± 96.8 μg/g). Firmicutes and Bacteroides populations also increased in FOS-consuming rats (Zheng et al. 2017). Sivieri et al. (2014) reported the prebiotic effect of FOSs as a growth enhancer of intestinal Lactobacilli and Bifidobacteria population. A simulator of the human intestinal microbial ecosystem (SHIME) model has been used in understanding the dynamics of the intestinal microflora using FOS (5 g/day) for 4 weeks. This resulted in a significant increase and selective enhancement in butyric and acetic acid levels. Synbiotic (short-chain FOS with *Lactobacillus fermentum*) action significantly increased *Bifidobacterium* population and acetic acid levels. A combination of *L. plantarum* and fructooligosaccharides was found to protect rural infants in India from neonatal sepsis (Panigrahi et al. 2017). Wong et al. (2019) reported the effect of Bifidobacterium breve M-16 V combined with prebiotic oligosaccharide mixture on atopic markers, to see the effect on *ex-vivo* cytokine production by peripheral blood mononuclear cells (PBMCs) and circulating regulatory T-cell percentage in infants with atopic dermatitis. Ale and Binetti (2021) demonstrated multiple effects of synbiotics in mouse models. Synbiotic administration resulted in increased mineral absorption due to production of SCFA, increase in the surface area of absorptive surface by promoting proliferation of enterocytes mediated by bacterial fermentation products, mainly lactate and butyrate, increased expression of calcium-binding proteins and degradation of mineral complexing phytic acid; release of the bone-modulating factors such as phytoestrogens, colonisation of the intestinal flora, stabilisation of the intestinal mucus, and impact of modulating growth factors such as polyamines (Abouloifa et al. 2020; Rahim et al. 2021).

### 7.2. Protection against colon cancer

Being dietary ingredients, the role of prebiotics in protection against the proliferation of colon cancer has been explored by many workers. The effect of neokestose was evaluated on cell proliferation, cell cycle and apoptosis of the colorectal cancer cell line (Caco-2) showed a significant and dose-dependent loss of viability of cancer cells (Lee et al. 2015; Faghfoori et al. 2021). A synbiotic mixture containing Yacon (*Smallanthus sonchifolius*) FOS and *L. acidophilus* CRL 1014 reduced the early phases of colon carcinogenesis in male Wistar rats (Almeida et al. 2015). *In vitro* study showed that neokestose inhibited the over-expression of nuclear factor-κB and cyclooxygenase-2 responsible for colorectal carcinoma. Neokestose can suppress the growth of melanoma cell lines (Wu et al. 2017). Li et al. (2017) demonstrated the metastatic effect of butyrate against colorectal cancer cells by deactivating signalling in a histone deacetylase-dependent manner. Reports suggested that lower rates of colorectal cancer occur in African counterparts as compared to Western countries due to the high use of unrefined fibre and high intakes of refined carbohydrates reported that propionate and butyrate have anti-inflammatory effects on colon cancer. The sugar fermentation activity is more suitable compared to protein hydrolysis because a sugar fermentation end product releases SCFA (acetate, propionate, and butyrate) (Chen and Vitetta 2018).

### 7.3. Improved mineral absorption

FOS imparts a positive effect on the absorption of minerals and hence can be of larger interest in the world scenario of mineral deficiency. Various mechanisms have been proposed to describe the possible role of FOS in mineral absorption improvement. Absorption of minerals generally occurs in the upper intestinal region and it has been observed that FOS consumption improved the mineral absorption process in the large intestine. The intake of SCFAs decreased pH with a concomitant increase in the production of butyrate and certain polyamines in the lumen. This environment is highly favourable for the absorption of mineral ions (Whisner and Castillo 2018; Azharia et al. 2021). The positive effect of FOS on calcium and magnesium absorption has been demonstrated in rats (Porwal et al. 2020). Another mechanism suggests the role of surface area enhancement with the colonic cell proliferation due to SCFA generation. FOS consumption led to an increase in cell permeability of the gut epithelium and promoted mineral absorption (Skrypnik and Suliburska 2017). FOS imparts a positive effect in the expression of transient receptor potential vanilloid (TRPV) genes, calbindin, and plasma membrane-bound calcium-ATPase (PMCA) involved in calcium absorption of rat colorectal mucosa cells by increasing TRPV mRNA expression (Ramírez-Barrantes et al. 2018).

### 7.4. Effect on serum lipid and cholesterol concentration

Serum lipids *viz*. cholesterol and triglycerides increase the risk of cardiovascular diseases which are a major public health concern. Food industries are highly interested in developing functional food ingredients to mitigate the risk of cardiovascular diseases by controlling serum lipids. Costa et al. (2015) reported the effect of FOS on insulin-resistant rats and their study suggested that daily FOS consumption enhanced HDL cholesterol levels and decreased LDL cholesterol and steatosis. Yu et al. (2020) reported that FOS supplementation effectively reduces hepatic steatosis, which confirmed a lipid-lowering effect on the model organism. FOS fermentation increases the SCFA in the intestine which helps to reduce the level of triglycerols and cholesterol, indicating the importance of FOS in managing hypercholesterolaemia. Insulin resistance and glucose homoeostasis are reported to be modulated by dietary fibres (Daguet et al. 2016; Yao et al. 2022).

### 7.5. Antioxidant property: Scavenging of free radicals

Dietary fibres are shown to have several physiological benefits to human health (Hua et al. 2021). Antioxidants are widely used as dietary supplements and have been investigated for the prevention of diseases such as cancer, coronary heart disease, and even altitude sickness. Picazo et al. (2019) have studied the effect of *Aspergillus oryzae* enzymatic extract on the properties of Aguamiel and after treatment, it was enriched with FOS which enhanced the growth of probiotic bacteria. FOS was produced as a result of *A. niger* Fasi treatment of *Arctium kappa* L. root extract and FOS enriched root extract showed high antioxidant activity (Tian et al. 2019). Purified GF2 and GF3 exhibited antioxidant activities as evidenced by ferric reducing antioxidant power (FRAP) and 1,1-diphenyl-2-picryl hydroxyl (DPPH) assay (Ojwach et al. 2020).

### 7.6. Dietary modulation of the human colonic microbiota

FOS stimulates and improves the growth of colonic microbiota (Bifidobacteria and Lactobacilli) by modifying the nutrient composition and also decreasing pathogenic bacteria and thus are highly useful as functional foods. These properties support the notion of FOS as a health-enhancing functional food ingredient. FOS is reported to be utilised as a selective fermentable substrate for Bifidobacteria and Lactobacilli. It has been pointed out that *L. acidophilus* and FOS supplementation can improve the health of the gut by removing certain faecal protein catabolites. The consensus statement of the International Scientific Association for Probiotics and Prebiotics (ISAPP) documents confirms that FOS-rich diets help in the growth of *Lactobacillus* and/or *Bifidobacterium* spp. more proficiently (Gibson et al. 2017; Dou et al. 2022). FOS and arabinogalactan administration to the simulator intestinal microbial ecosystem resulted in an increase in SCFA production in different colon areas (Daguet et al. 2016). Rigo-Adrover et al. (2018) reported the effect of FOS, galacto-oligosaccharides, fermented milk, and pectin-derived oligosaccharides containing diet can protect suckling rats from rotavirus gastroenteritis. The report showed that the amount of Bifidobacteria in the oligosaccharide-treated rats was significantly healthier compared to the controls. Furthermore, some studies have confirmed that pregnancy to early life is the prime time for the colonisation of the infant intestinal microflora (Yao et al. 2021). Studies suggest that the mechanism of action of prebiotics in gut ecosystem modification for digestive disorders management is through interactions with both the host and the microbiome *via* molecular effectors present on the cell structure and generated metabolic products. Probiotic metabolites act on the microbiota through cross-feeding interactions, changes in the gastrointestinal microenvironment (pH lowering), competition for nutrients and binding sites, and inhibition of growth via the production of strain-specific antibacterial compounds including bacteriocins (Plaza-Diaz et al. 2019; Cunningham et al. 2021).

### 7.7. Immuno-modulatory effect

Fermentation products of FOS interact with and affect the gut-associated lymphoid tissue (GALT) and systemic immune system (Yoo et al. 2020). Several reports suggest that scFOS supplementation influences intestinal immune function and imparts beneficial effects (Zhao et al. 2019; Wu et al. 2020). Clinical trial studies confirmed the role of probiotics, prebiotics, postbiotics, and omega-3 in promotion of the intestinal health and these substances can reduce the level of cytokines, interleukin, clinical inflammation, and necrosis factor in ulcerative colitis (Plaza-Diaz et al. 2019). Similar effects of FOS and inulin are reported in patients with Crohn’s disease where it improved the amount of mucosal dendritic cells (IL10 positive) (Maioli et al. 2022). The FOS-enriched diet may increase the total cell yield and B-lymphocytes. In contrast, the effect of FOS enrichment increased the level of T-lymphocytes in lipopolysaccharides (LPS) challenged mice, while it remained unchanged in healthy mice. Various reports are suggesting the immuno-modulation effect of FOS (Rigo-Adrover et al. 2018; Young et al. 2021; Zhang et al. 2021). Hachimura et al. (2018) described some of the mechanisms involved in immunomodulation by FOS rich diet. FOS selectively enhanced certain gut microbial populations which, in turn, induced the secretion of IL-6 or TGF-β from dendritic cells.

## 8. Conclusions

The biotechnological application of fungal FTases plays a significant role in the generation of prebiotic FOS. The industrial production of food-grade oligosaccharides is expanding day by day due to its pharmaceutical and nutritional importance. Commercial production of FOS is achieved by FTases which transfructosylate sucrose to generate GF4, GF3, and GF2. To meet the increasing demand, FTase production strategies are engaging novel fungal isolates and optimised fermentation protocols. Fungal FTases have been heterologously expressed in yeast and bacterial expression systems for their efficient production. Pertaining to its increasing use in beverages, drugs, feed, nutraceuticals, and infant formula foods, the FOS market is growing at a CAGR of 8.8% and is expected to reach 5.09 billion USD by 2030. FOS-generating processes based on fungal FTases, therefore, have an important role to play in the coming times.
